# Giant hepatic hemangioma with internal necrosis discovered due to fever of unknown origin and treated successfully with surgical resection: a case report

**DOI:** 10.1007/s12328-025-02145-8

**Published:** 2025-05-22

**Authors:** Rei Ryozawa, Hirohito Takeuchi, Katsutoshi Sugimoto, Hiroaki Osakabe, Chie Takishita, Jun Matsubayashi, Yuichi Nagakawa, Toshitaka Nagao, Takao Itoi

**Affiliations:** 1 Department of Gastroenterology and Hepatology, Tokyo Medical University, 6-7-1 Nishishinjuku, Shinjuku-ku, Tokyo 160-0023 Japan; 2 Department of Gastrointestinal and Pediatric Surgery, Tokyo Medical University, Tokyo, Japan; 3 Department of Pathology, Tokyo Medical University, Tokyo, Japan

**Keywords:** Giant cavernous hepatic hemangioma, Hepatectomy, Fever

## Abstract

Hepatic hemangiomas are typically asymptomatic benign liver tumors. This report describes a case of a large hepatic hemangioma with internal bleeding and necrosis, presenting as fever of unknown origin, which was successfully treated with surgical resection. A woman in her 40s presented with persistent fever and fatigue. Imaging revealed a 13 cm mass in the posterior sector of the right hepatic lobe, with areas of high attenuation suggestive of internal bleeding. Laboratory tests revealed elevated levels of C-reactive protein, interleukin-6, and complement components (C3, C4, and CH50), along with an increased erythrocyte sedimentation rate. Symptomatic treatment with antipyretic medications failed to resolve the fever; therefore, hepatic resection was performed for diagnostic and therapeutic purposes. Post-operative recovery was uneventful, and the fever resolved completely. Pathological examination revealed cavernous hemangioma with well-defined necrotic areas. Post-operative blood tests showed normalization of the preoperatively elevated prognostic markers. Bleeding and necrosis associated with a large hemangioma appear to trigger the release of damage-associated molecular patterns, stimulating interleukin 6 production, promoting prostaglandin E2 synthesis, and ultimately leading to fever. Hepatic resection is an effective treatment for large hemangiomas in patients presenting with fever.

## Introduction

Hepatic hemangiomas are common benign liver tumors, accounting for 73% of all benign hepatic tumors [1]. They are present in 0.4–20% of the population and occur most frequently in women aged 30–50 years [2, 3]. Hemangiomas consist of blood-filled cavities lined with endothelial cells and are supplied by branches of the hepatic artery [4]. Their development may be influenced by congenital anomalies, abnormal vascular formation, and hormones, such as estrogen. Symptomatic giant hemangiomas (>10 cm) with bleeding, rupture, or necrosis require further treatment. The first-line treatment is surgical resection, including hepatectomy, enucleation, laparoscopy, or robotic techniques. Other treatment options include liver transplantation, radiofrequency ablation, transarterial embolization (TAE), radiotherapy, and chemotherapy. This report describes a case of a large hepatic hemangioma with internal bleeding and necrosis, presenting as fever of unknown origin, which was successfully treated with surgical resection.

## Case report

**Fig. 1:**
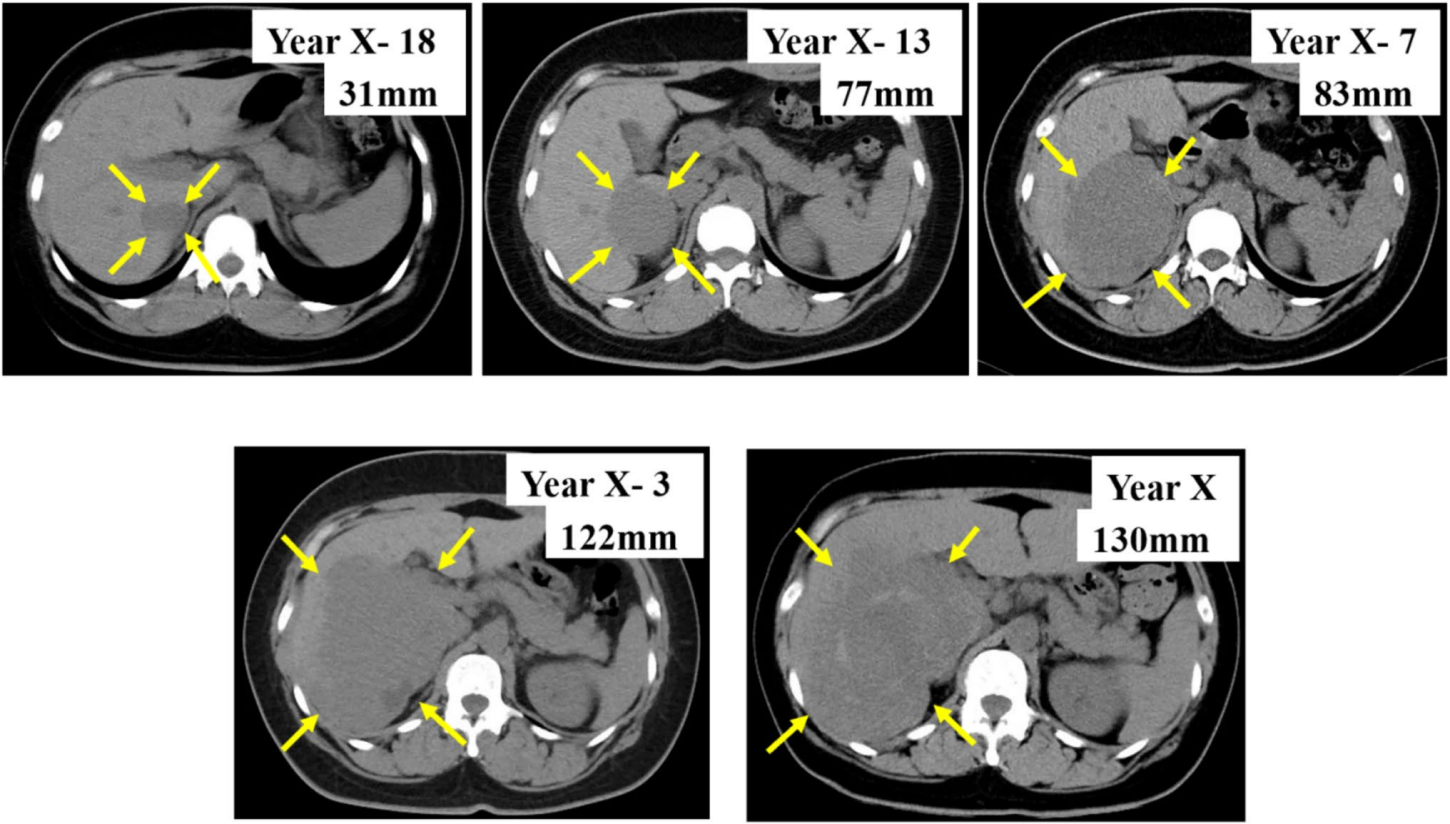
Abdominal CT. The hepatic hemangioma in the posterior zone of the right lobe of the liver showed an increased transition from 31 mm to 130 mm over the course of 18 years. The yellow arrows indicate the edge of hepatic hemangioma. *CT* computed tomography

**Fig. 2:**
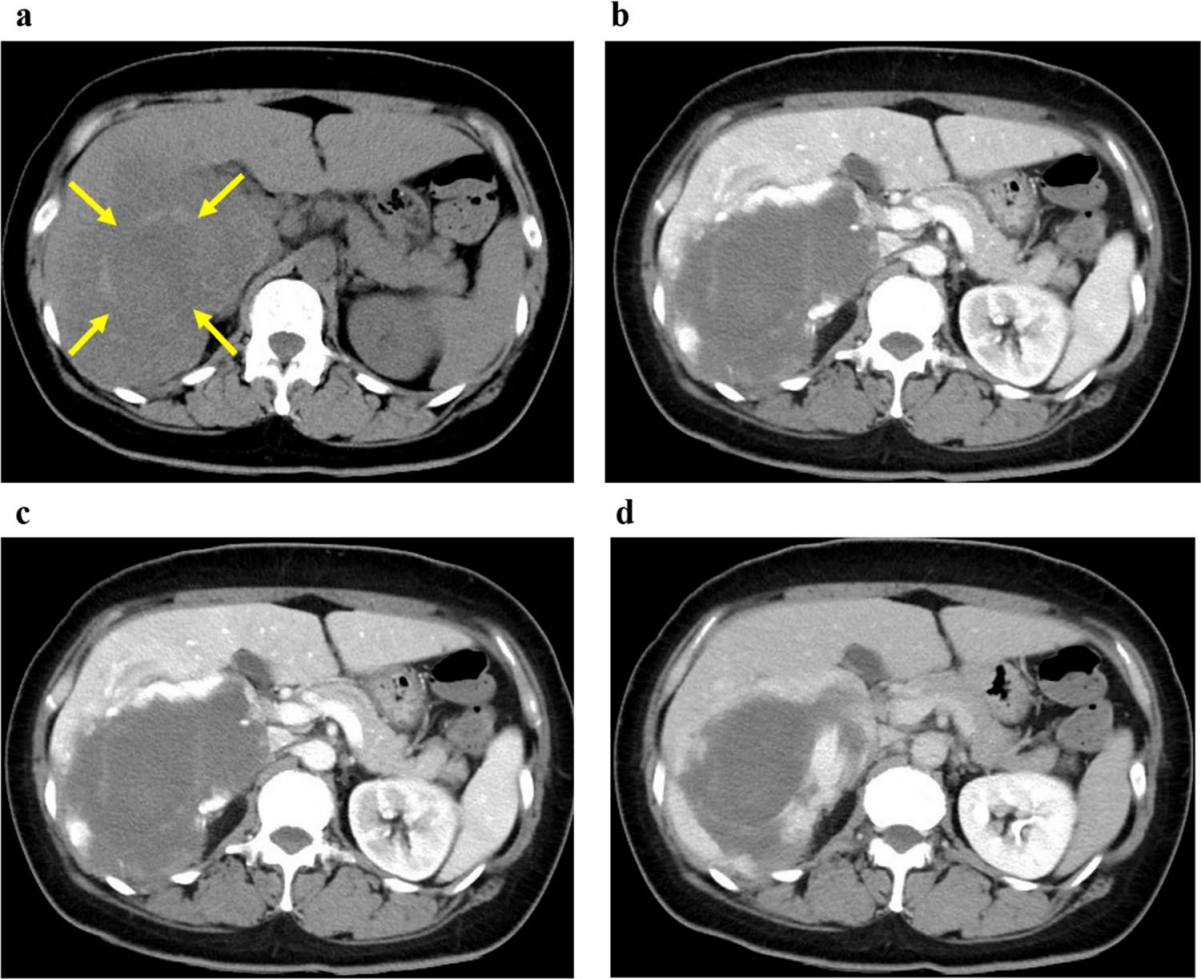
Abdominal contrast-enhanced CT. **a** Plain. **b** Artery phase. **c** Portal phase. **d** Delay phase **d**. A high-density area (indicated by yellow arrows) suspicious of hemorrhage and necrosis inside the giant hemangioma, was observed. *CT* computed tomography

**Fig. 3:**
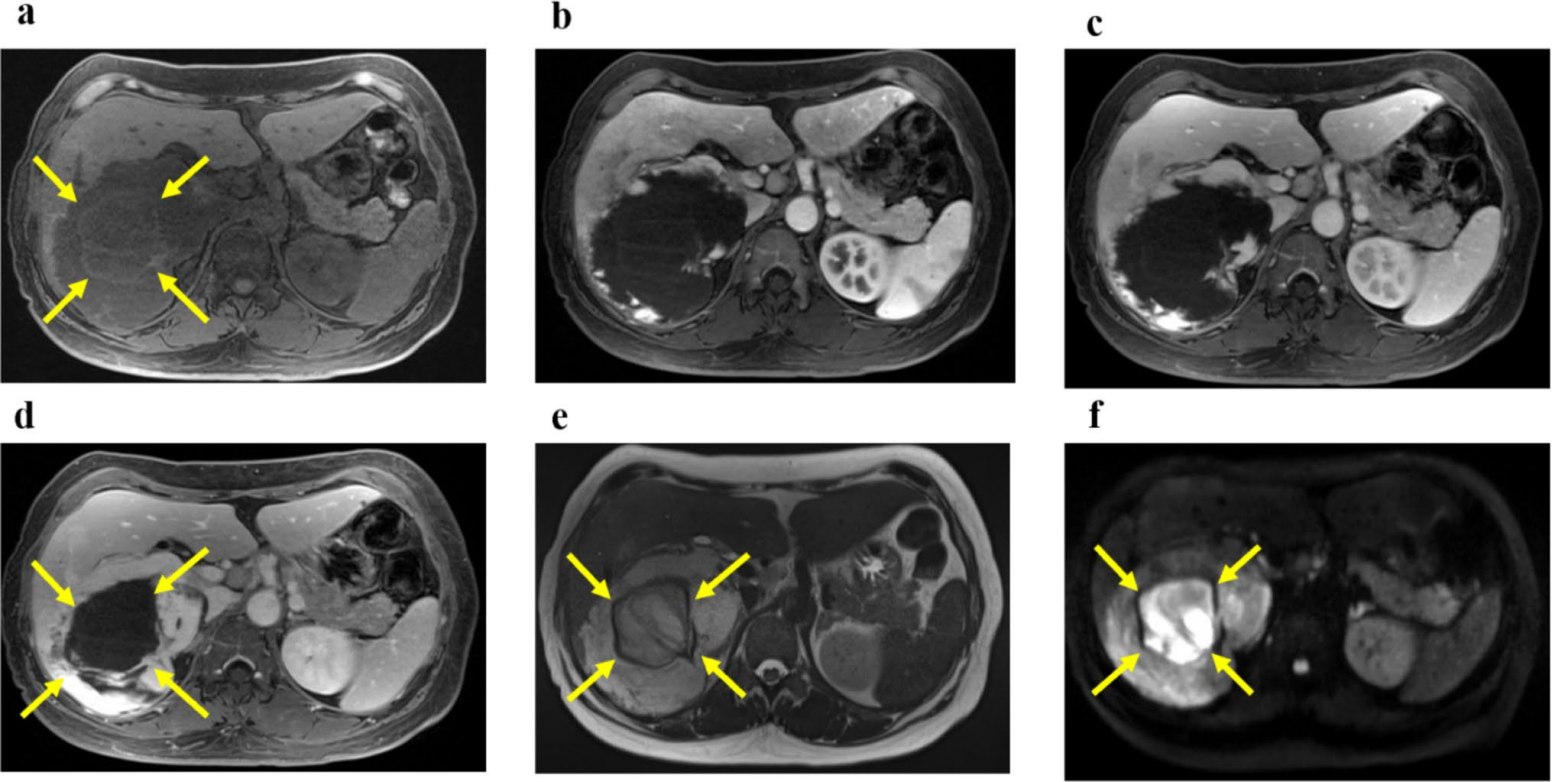
Abdominal MRI. T1 weighted-enhanced images **a**. Artery phase **b**. Portal phase **c**. Delay phase **d**. T2 weighted-enhanced image **e**. Diffusion-weighted image b=800 **f**. A giant hemangioma, 130 mm in size, was observed in the posterior right lobe of the liver and contrasted with the periphery. The interior of the hemangioma showed a well-defined non-contrast area, which appeared as an abnormal signal area in the diffusion-weighted image. MRI, magnetic resonance imaging. The yellow arrows indicate areas of hemorrhage and necrosis within the giant hemangioma

**Fig. 4:**
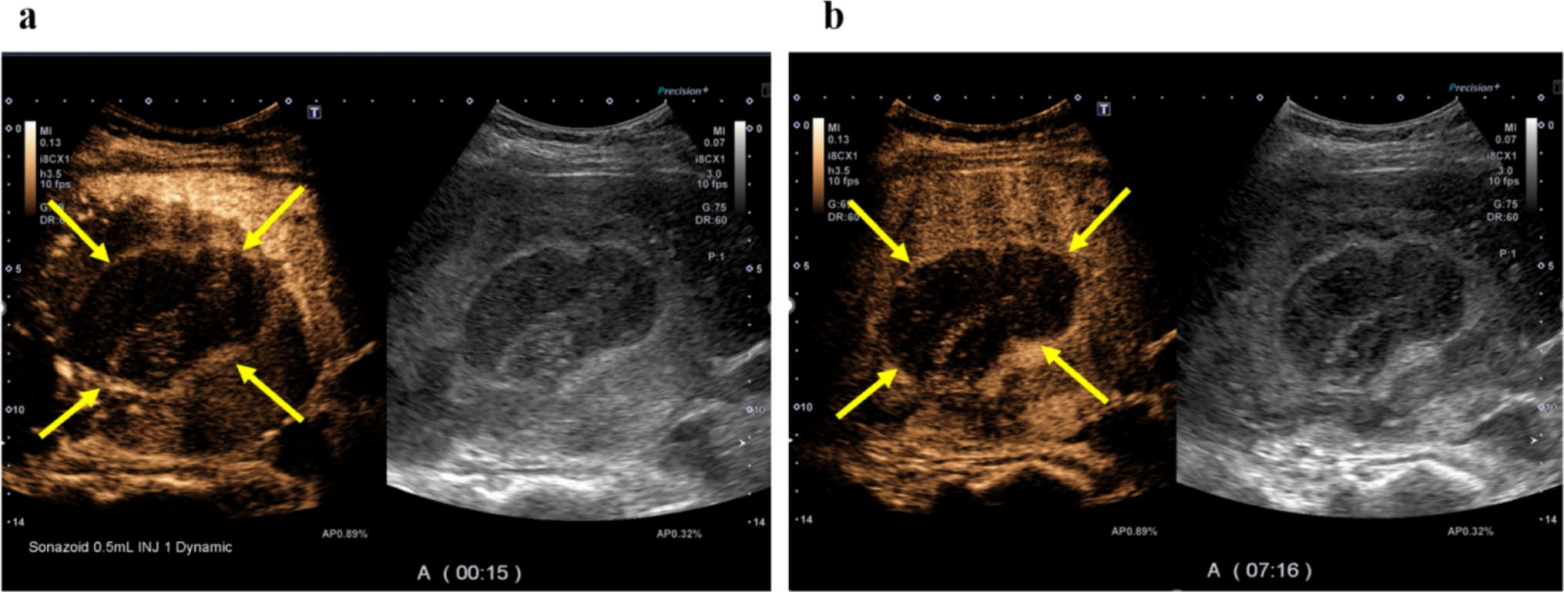
Abdominal contrast-enhanced ultrasound. **a** Vascular imaging and **b** Kupffer imaging. The arterial phase after injection of ultrasound contrast medium showed a fill-in pattern with contrast from the periphery of the hemangioma. Kupffer imaging showed a well-defined, non-contrasted area inside the hemangioma. The yellow arrows indicate it

**Fig. 5:**
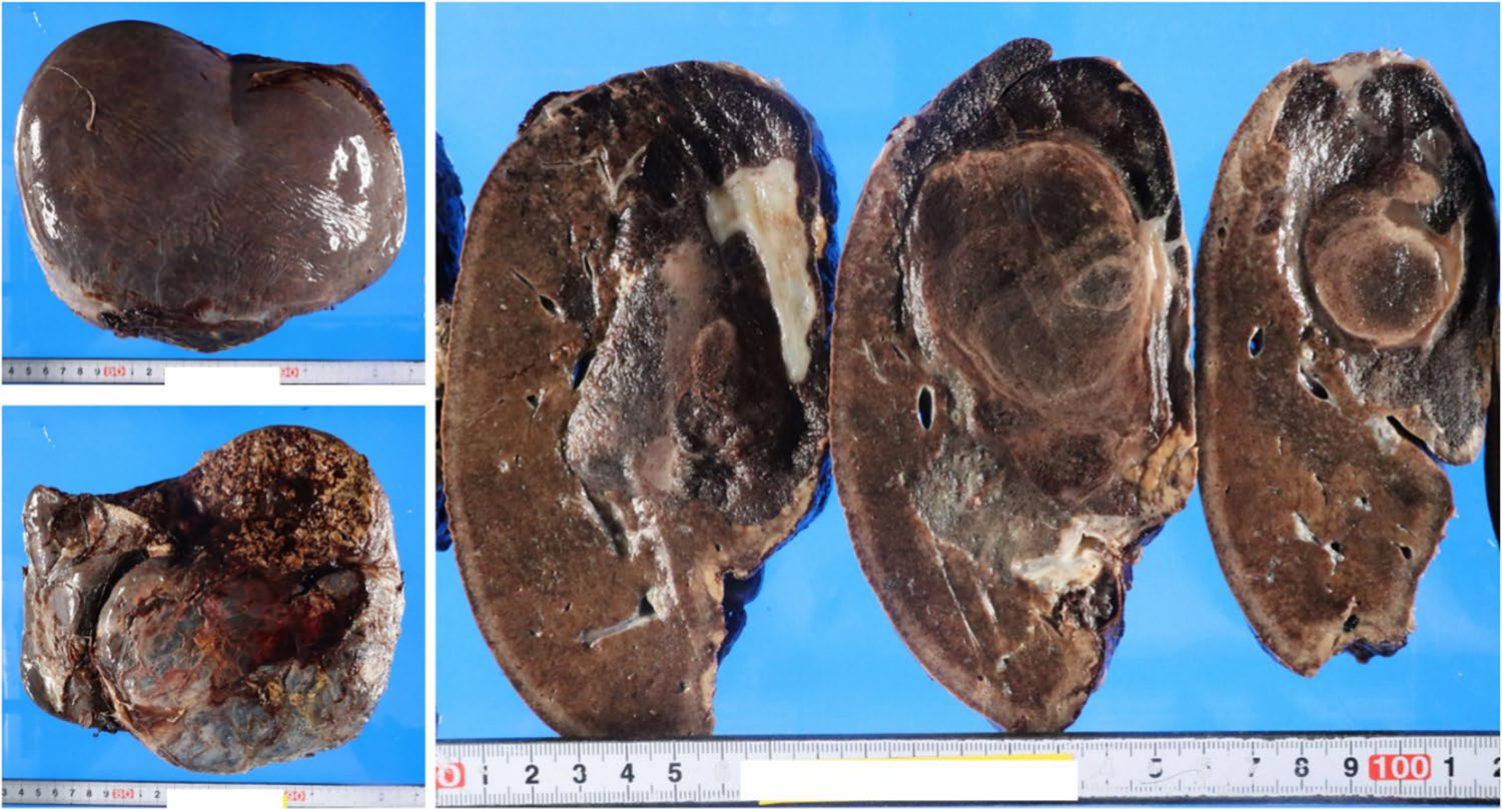
Grossly, the excised mass appeared to be a well-circumscribed dark reddish mass with a thin fibrous capsule (140×130×90 mm)

**Fig. 6:**
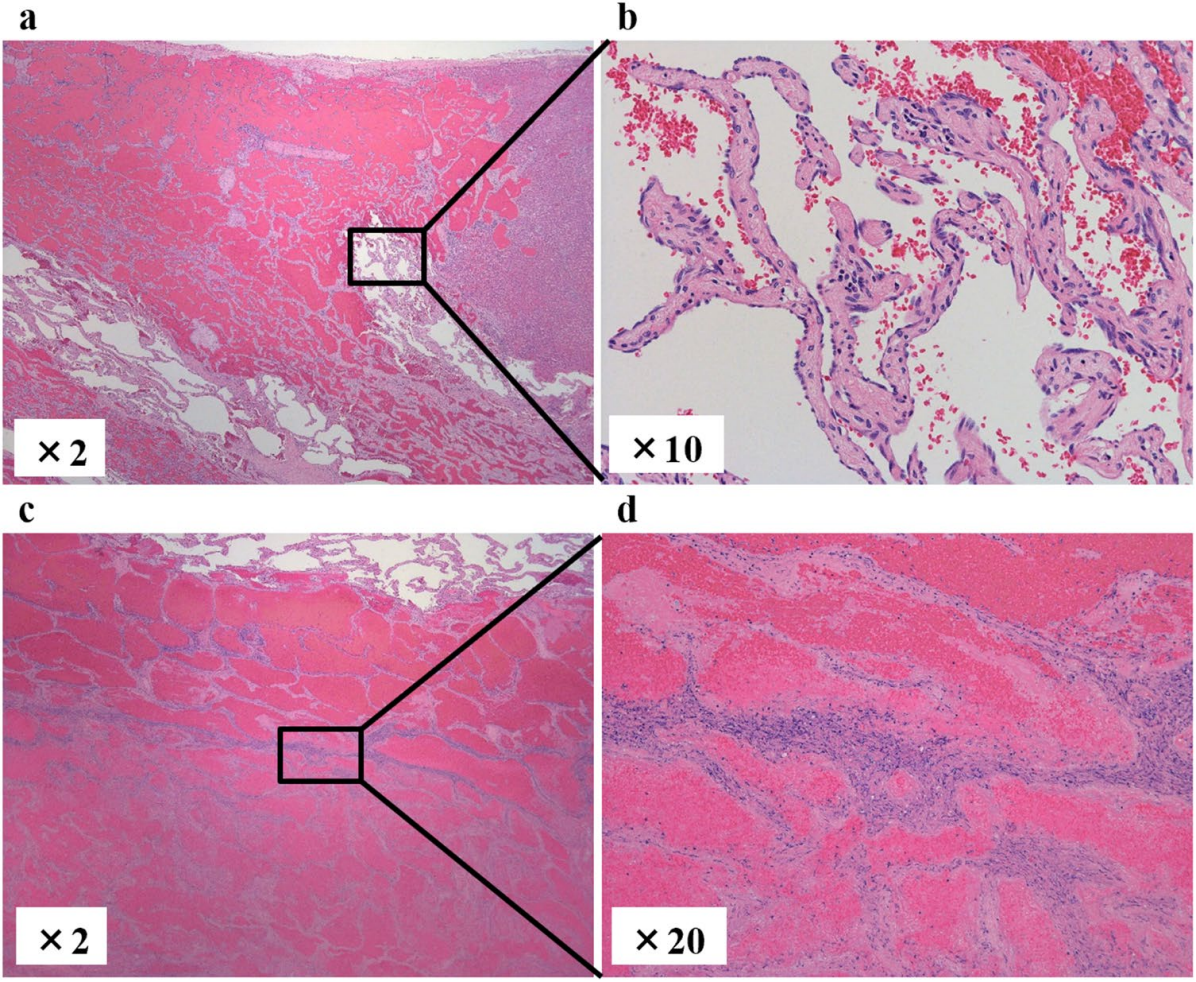
Microscopic pathological findings of the liver. (**a**, **b**) The tumor comprised dilated, cavernous vascular spaces lined by a single layer of endothelial cells, and there was a nearly acellular fibrous matrix in the walls of the cavernous spaces. (**c**, **d**) The central area of the tumor was necrotic, and nuclear debris at the margins of the necrotic area was observed

**Fig. 7:**
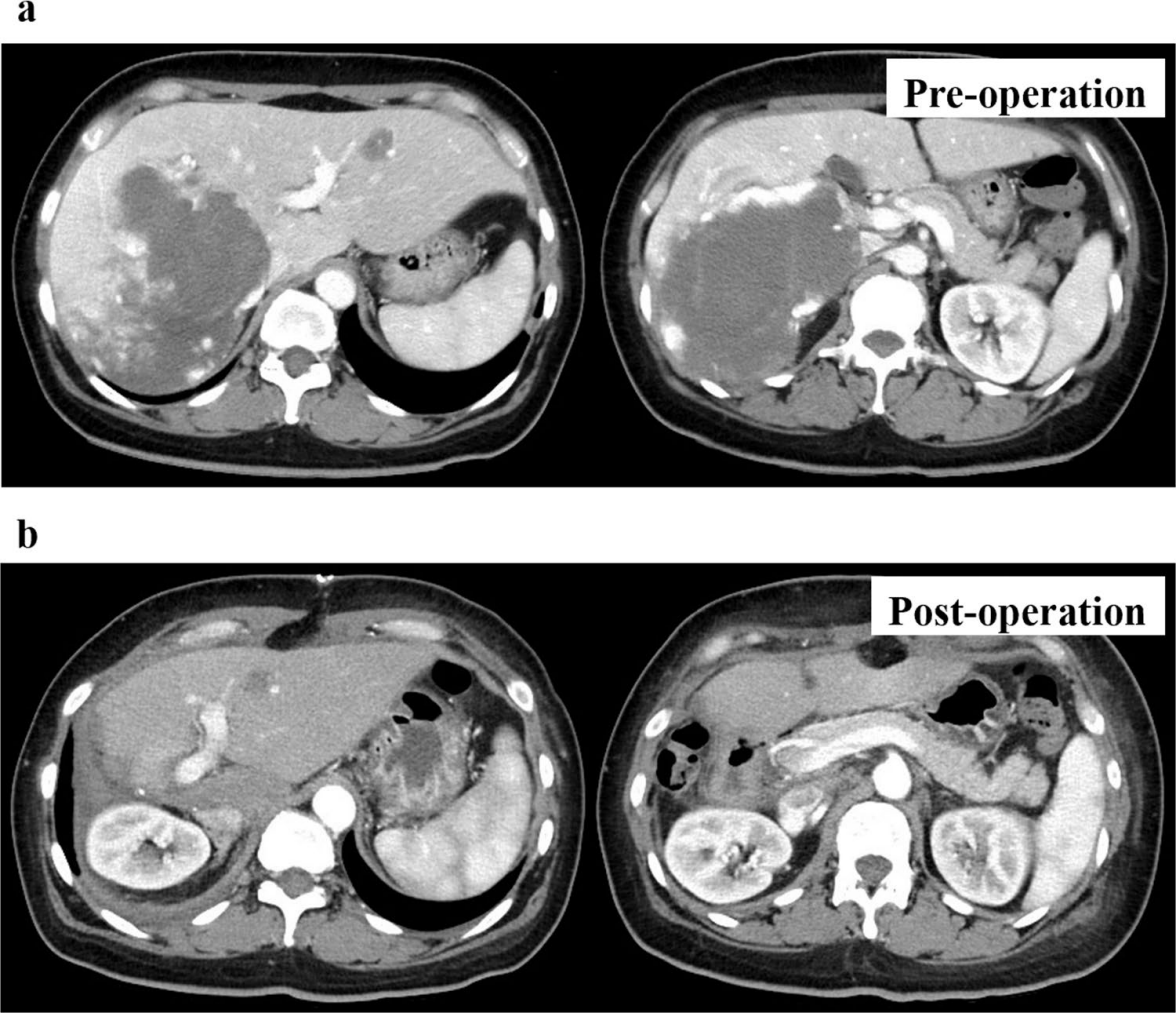
Abdominal contrast-enhanced CT (arterial phase). **a** Pre-operation. **b** Post-operation. The giant hepatic hemangioma was successfully removed, and no complications were associated with the surgery. *CT* computed tomography

A woman in her 40s presented with persistent fever and fatigue. A hepatic hemangioma measuring 3 cm in the posterior sector of the right hepatic lobe had been identified on abdominal computed tomography (CT) approximately 20 years earlier. Three years prior, its size had increased to 12 cm (Fig. 1). Considering that the rupture of the giant hepatic hemangioma could result in massive bleeding and hemorrhagic shock, surgery could have been considered once the size exceeded 10 cm. However, surgery or other treatment was not administered as she did not experience any abdominal pain, tenderness, or fever until after giving birth. Annual follow-ups with non-contrast magnetic resonance imaging (MRI) were initiated. On December X–1, she gave birth to her first child and had no fever prior to childbirth. Nine days postpartum, she developed persistent chills and fever. Despite antibiotic and symptomatic treatment, her symptoms persisted, leading to her visit to our hospital on January X. Her medical history included total thyroidectomy for thyroid cancer in her 20s, for which she had been receiving post-operative levothyroxine. Physical examination upon admission revealed the following: height, 150.2 cm; weight, 67.5 kg; body temperature, 38.3 ℃; blood pressure, 110/80 mmHg; pulse, 90 bpm; and oxygen saturation, 99% on room air. Abdominal examination revealed a soft, flat abdomen without tenderness or pain. Table 1 shows the blood test results. Laboratory findings revealed the following: white blood cell count, 6,800/μL (); C-reactive protein (CRP), 10.36 mg/dL (elevated); aspartate aminotransferase, 53 U/L; alanine aminotransferase, 63 U/L; alkaline phosphatase, 295 U/L (mildly elevated); hemoglobin, 8.9 g/dL (mild anemia); coagulation parameters, normal; elevated IL-6, C3, C4, and CH50 levels; and increased erythrocyte sedimentation rate (ESR). Abdominal contrast-enhanced CT revealed a 13 cm giant mass in the posterior segment of the right hepatic lobe, with hyperdense areas suggestive of hemorrhage (Fig. 2). MRI revealed abnormal signal intensity in the tumor center with a gradual enhancement pattern from the periphery (Fig. 3). Ultrasonography revealed a large, heterogeneous, hyperechoic mass with low-echo central regions. Contrast-enhanced ultrasound showed a “fill-in” pattern and an avascular center, suggesting necrosis (Fig. 4). Differential diagnoses included hemorrhage and necrosis within the hemangioma, postpartum-related conditions, collagen disease, vasculitis, infection, and the effects of prior thyroid cancer treatment. Based on imaging and laboratory findings, the fever was attributed to hemorrhage and necrosis of the hemangioma. Hepatectomy was performed because of the patient’s preserved hepatic function (ICG 5.0%) and her preference for definitive treatment. Open surgery was performed after confirming the location of the hepatic hemangioma by ultrasound, and the liver parenchyma, including the hepatic hemangioma, was removed. The surgery lasted 7 h, with minimal blood loss, and transfusion was not required. Pathological examination ruled out malignancies such as angiosarcoma or hepatic abscess. Macroscopically, the excised mass measured 140×130×90 mm and appeared to be a well-circumscribed dark reddish mass with a thin fibrous capsule (Fig. 5). Microscopically, the tumor comprised dilated cavernous vascular spaces lined with a single layer of endothelial cells, with a nearly acellular fibrous matrix in the walls of the cavernous spaces. The central area of the tumor was necrotic, and nuclear debris was observed at the margins of the necrotic area (Fig. 6). Gram stain showed no fungal colonies and no bacteria phagocytosed by neutrophils or macrophages. Figure. 7 illustrates the comparison between pre- and post-operative liver images on abdominal contrast-enhanced CT. The giant hepatic hemangioma was successfully removed, and there were no complications associated with the surgery. Postoperatively, the patient recovered well, and her fever resolved. CRP levels decreased from 10.36 mg/dL to 0.79 mg/dL, and IL-6 normalized from 47.6 pg/mL to 3.2 pg/mL. The white blood cell count decreased from 6800/μL to 3900/μL. Complement levels (C3, C4, and CH50) and ESR also returned to normal.

**Table 1:** Blood test

Hematology		Tumor markers	
WBC	6800/μL	AFP	2.0 ng/mL
Neut	83.4%	AFP-L3	<5.0%
Lymph	8.8%	PIVKA-II	17 mAU/mL
Mono	7.7%	CEA	<2.0 ng/mL
Eos	0.0%	CA19-9	5.7 U/mL
Baso	0.1%	CA125	14.8 U/mL
RBC	3.24×10^4^/μL		
Hb	8.9 g/dL	**Serology**	
Ht	29.0%	IgG	2005.0 mg/dL
PLT	52.8×10^4^/μL	IgM	68.0 mg/dL
		IgA	338.0 mg/dL
**Biochemistry**		IgG4	27.9 mg/dL
TP	7.4 g/dL	ANA	<40
ALB	2.5 g/dL	RF	7.1 U/mL
T-BIL	0.26 mg/dL	DNA antibody	<10 IU/mL
AST	53 U/L	SS-A antibody	≥1200 U/mL
ALT	63 U/L	CLβ2GP1 antibody	≤1.2 U/mL
ALP	295 U/L	Lupus anticoagulant	1.2
GGT	127 U/L	Anti-cardiolipin immunoglobulin G	1.2 U/mL
LDH	175 U/L	IL-6	47.6 pg/mL
BUN	8.2 mg/dL	C3	177 mg/dL
Cr	0.60 mg/dL	C4	43 mg/dL
Amylase	64 U/L	CH50	78.0 mg/dL
Lipase	28.6 U/L	TSH	0.06 μIU/mL
NH₃	49 μg/dL	FT3	2.11 pg/mL
CRP	10.36 mg/dL	FT4	2.14 ng/mL
Ferritin	423.4 ng/mL	β-D-glucan	<6.0 pg/mL
		Endotoxin	<3.5 pg/mL
**Coagulation**		Procalcitonin	0.19 ng/mL
PT-INR	1.10		
PT control	12.8	**Hepatitis virus**	
PT	14.0 s	HBs antigen	(–)
APTT control	28.6	HCV antibody	(–)
APTT	36.9 s	TPLA	(–)
FDP	5.1 μg/mL		
D-dimer	2.31 μg/mL		
Fibrinogen	700 mg/dL		
AT-III	92.5%		

*WBC* white blood cell count, *AFP* alpha-fetoprotein, *Neut* neutrophil percentage, *AFP-L3* alpha-fetoprotein L3 isoform, *lymph* lymphocyte percentage, *PIVKA-II* protein induced by vitamin K absence or antagonist-II, *Mono* monocyte percentage, *CEA* carcinoembryonic antigen, *Eos* eosinophil percentage, *CA19-9* cancer antigen 19-9, *Baso* basophil percentage, *CA125* cancer antigen 125, *RBC* red blood cell count, *Hb* haemoglobin, *Ht* haematocrit, *PLT* platelet count, *TP* total protein, *ALB* albumin, *T-BIL* total bilirubin, *AST* aspartate aminotransferase, *ALT* alanine aminotransferase, *ALP* alkaline phosphatase, *GGT* gamma-glutamyl transferase, *LDH* lactate dehydrogenase, *BUN* blood urea nitrogen, *Cr* creatinine, *CRP* C-Reactive protein, *PT-INR* Prothrombin time—international normalized ratio, *APTT* activated partial thromboplastin time, *FDP* fibrin degradation products, *IL-6* Interleukin-6, *CH50* total hemolytic complement, *TSH* thyroid-stimulating hormone, *HB* hepatitis B, *HCV antibody* hepatitis C virus antibody, and *TPLA* thromboplastin antibody.

## Discussion

The giant hepatic hemangioma in this case resulted in internal necrosis and fever of unknown etiology. Histopathological findings revealed coagulation necrosis at the center of the mass, suggesting infarction or circulatory disturbance. Additionally, hepatocytes surrounding the necrotic area exhibited marked congestion. The presence of neutrophils, monocytes, and fibrin deposition in the border zone suggests that extensive infarction occurred within the hepatic hemangioma and that an inflammatory reaction may have occurred at the infarct margin. In the present case, fever and an elevated inflammatory response were observed a few days after delivery of the first child, suggesting that physical pressure, such as that from the uterus, may have caused impaired blood flow in the inflow artery. This likely triggered an immune response, leading to the release of inflammatory mediators such as IL-6, which subsequently induced fever. Previous case reports of hepatic hemangiomas with fever have described varying pathological findings. Among 13 reported cases, 6 exhibited internal hemorrhage alone, 4 exhibited necrosis alone, and 2 exhibited internal hemorrhage and necrosis (Table 2) [5–14]. In one case, positive staining for IL-6 was detected in sinusoidal endothelial cells and inflammatory infiltrates [6]. However, no studies have reported the measurement of IL-6 levels. In this case, IL-6 levels were measured before and after surgical treatment, confirming rapid improvement after surgery. Furthermore, the white blood cell count, CRP, C3, C4, CH50, and ESR improved, suggesting that the unknown fever might have been caused by the necrotic area of the hepatic hemangioma. IL-6 is known to be induced not only by bacterial infections [15] but also by stimulation from damage-associated molecular patterns (DAMPs) released from injured or dead cells. This stimulation can trigger an inflammatory response, ultimately leading to fever [16, 17]. When tissue damage, such as necrosis or hemorrhage, occurs, DAMPs stimulate IL-6 release. IL-6 plays a key role in regulating acute-phase responses, mobilizing leukocytes, and inducing fever. Specifically, IL-6 acts on the hypothalamus to stimulate the production of prostaglandin E2 (PGE2), which affects the thermoregulatory center and induces fever. This mechanism helps increase body temperature during tissue damage or infection, promotes pathogen suppression, and enhances immune responses [17]. The chronic elevation of IL-6 levels in blood tests may indicate persistent inflammation or tissue damage, necessitating appropriate diagnosis and treatment. Hepatic hemangiomas are typically benign tumors that remain asymptomatic even when large. However, internal bleeding or necrosis can lead to symptoms such as fever, complicating the diagnosis and resulting in classification as fever of unknown origin. In this case, the patient had a performance status (PS) of 0 and normal liver function, with an indocyanine green retention rate of 5% at 15 min (ICG15), making surgical resection an effective treatment. Unknown fever caused by necrosis within a hepatic hemangioma has been reported to persist for 1–11 months [6]. In the present case, the patient experienced persistent fever exceeding 38 °C and elevated CRP levels for over one month, accompanied by a progressive decline in albumin levels, indicative of wasting. We considered steroid administration or hepatic arterial embolization as treatment options; however, these findings highlight the importance of surgical treatment. In cases where surgical resection is not viable, anti-inflammatory treatments such as steroids or NSAIDs are used for symptomatic management. Given that IL-6 plays a central role in inflammation, IL-6 inhibitors represent a potential therapeutic option. Measuring IL-6 levels and utilizing imaging diagnostics in similar cases can facilitate early and appropriate treatment.

**Table 2:** Cases of hepatic hemangioma causing fever

Ref.	Age	Sex	Tumor size	Inside the hepatic hemangioma	IL-6 stain	Treatment	Outcome
5	38	F	Right lobe of the liver	Hemorrhage	–	Right hepatectomy	Cured
6	53	F	10 cm	Internal hemorrhage, hematoma	Positive staining	Hepatic resection	Cured
7	45	F	Giant, left lobe	Internal hemorrhage	–	–	–
8	43	F	Entire right lobe	Internal hemorrhage	–	Right hepatic lobectomy/ prednisone	Cured
9	46	F	18×14 cm	–	–	Right hepatic lobectomy	Cured
10	47	M	Giant, right lobe	Necrosis	–	Right hepatectomy	Cured
	44	M	Giant, left lobe	Necrosis	–	Left hepatectomy	Cured
11	50	M	Left lobe of the liver	–	–	Surgical resection of the mass	Cured
12	49	F	15×11 cm	Necrosis	–	Laparoscopic-assisted left lateral segmentectomy	Cured
13	32	M	20 cm	Necrosis	–	Right trisectionectomy	Cured
14	59	F	9×6.6×10 cm	Infected necrosis, internal hemorrhage	–	Hepatic resection	Cured
Our case	44	F	13 cm	Internal hemorrhage, necrosis	–	Right hepatectomy	Cured

*F* female, *M* male
